# Evaluation and trend of fashion design research: visualization analysis based on CiteSpace

**DOI:** 10.1186/s40691-022-00316-6

**Published:** 2022-12-25

**Authors:** Yixin Zou, Sarawuth Pintong, Tao Shen, Ding-Bang Luh

**Affiliations:** 1 School of Art and Design, Guangdong University of Technology, Guangzhou, 510062 China; 2 Culture-Based Design Arts Program, Faculty of Decorative Arts, Silpakorn Univeristy, Bangkok, 10170 Thailand; 3 College of Design and Innovation, Tongji University, Shanghai, 200092 China

**Keywords:** Bibliometric, CiteSpace, Fashion, Fashion design, Knowledge mapping

## Abstract

Fashion or apparel refers to a topic discussed publicly as an indispensable discipline on a day-to-day basis, which has aroused rising attention from academic sessions over the past two decades. However, since the topic of fashion design covers knowledge in extensive ranges and considerable information, scholars have not fully grasped the research field of fashion design, and the research lacks directional guidance. To gain more insights into the existing research status and fronts in the fashion design field, this study conducts a quantitative literature analysis. The research of this study is conducted by employing CiteSpace technology to visualize and analyze 1388 articles regarding “fashion design” in the Web of Science (WOS) Core Collection. To be specific, the visualization and the analysis concentrate on the annual number of articles, author collaboration, institutional collaboration, literature citations, keywords clustering, and research trend evolution of the mentioned articles. As highlighted by this study, the effect of the US and the UK on academic research in fashion design is relatively stronger and extensive. Sustainable fashion refers to the research topic having aroused more attention since 2010, while new research topics over the past few years consist of “wearable fashion”, “transgender fashion” and “medical fashion”. The overall research trend of fashion design is developing as interdisciplinary cross research. This study systematically reviews the relevant literature, classifies the existing research status, research hotspots and frontier trends in the academic field of “fashion design”, and presents the knowledge map and information of literature for researchers in relevant fields.

## Introduction

In academic research and writing, researchers should constantly search relevant literature to gain systematic insights into the subject area (e.g., the major research questions in the field, the seminal studies, the landmark studies, the most critical theories, methods and techniques, as well as the most serious current challenges). The process to answer the mentioned questions refers to an abstract process, which requires constant analysis, deduction and generalization. Any literature emerging over time may be critical, any research perspective may cause novel inspiration, and any detail can be the beginning of the subsequent research. However, when literature is being sorted and analyzed, if judgment only complies with personal experience, important literature will be inevitably missed, or the research direction will be lost in the research. For the process of conducting literature analysis, Hoover proposed that the quantitative methods of literature represent elements or features of literary texts numerically, applying effective, accurate and widely accepted mathematical methods to measure, classify and analyze literature quantitatively (Hoover, 2013). On this basis, literary data and information are more comprehensively processed. Prof. Chaomei Chen developed CiteSpace to collect, analyze, deliver and visualize literature information by creating images, diagrams or animations, thereby helping develop scientific knowledge maps and data mining of scientific literature. Knowledge visualization primarily aims to detect and monitor the existing state of research and research evolution in a knowledge field. Knowledge visualization has been exploited to explore trends in fields (e.g., medical, management science, biomedicine and biotechnology).

However, the international research situation in fashion design has not been analyzed by scholars thus far. Fashion, a category of discourse, has been arousing scholars’ attention since the late nineteenth century (Kim, 1998). In such an era, fashion is recognized by individuals of all classes and cultures, and it is publicly perceived. The field of fashion design is significantly correlated with people's lives (Boodro, 1990), and numerous nations and universities have long developed courses regarding fashion design or fashion. Besides, the development of fashion acts as a symbol of the soft power of the country. The discussion on fashion trend, fashion designer, fashion brands, artwork and other topics in the society turns out to be the hotspot discussed on a nearly day-to-day basis, and the discussion in the society even exceeds the academic research. However, the academic research of fashion design refers to a topic that cannot be ignored. The accumulation and achievements of academic research are manifested as precipitation of knowledge for developing the existing fashion field, while significantly guiding future generations. Studying the publishing situation and information in fashion design will help fashion practitioners or researchers classify their knowledge and provide them with novel inspiration or research and literature directions.

This study complies with the method of quantitative literature analysis, and CiteSpace software is adopted to analyze the literature in fashion design. Through searching web of science (WOS) Core Collection, 1388 articles regarding “fashion design” are retained. Co-citation, co-authoring and co-occurrence analysis refer to the major functions of CiteSpace. This study analyzes the articles regarding “fashion design”, and the focus is placed on the annual publication volume, author collaboration, institutional collaboration, national collaboration, literature citations, keyword co-occurrence, keyword clustering, and the research evolution, and visualized the literature and research as figures of these articles. The results here are presented as figures. This study provides the fronts knowledge, the current research status research, the hotspots and trends in fashion design research.

## Methods

In this study, CiteSpace technology is adopted to analyze all collected literature data. CiteSpace, developed by Professor Chaomei Chen, an internationally renowned expert in information visualization at Drexel University, USA (Wang & Lu, 2020), refers to a Java application to visually analyze literature and co-citation networks (Chen, 2004). CiteSpace is capable of displaying burst detection, mediated centrality and heterogeneous networks regarding literate information. Visual analysis of the literature by using CiteSpace covers three functions, i.e., to identify the nature of specialized research frontiers, to label and cluster specialized research areas, as well as to identify the research trends and abrupt changes based on the data derived from the analysis. CiteSpace provides a valuable, timely, reproducible and flexible method to track the development of research trends and identify vital evidence (Chen et al., 2012).

To analyze the existing status of research and publications on the topic of “fashion design” in academia and different nations, the “Web of Science” (WOS) database is adopted as the data collection source here. Web of Science provides seamless access to existing and multidisciplinary information from approximately 8700 of the most extensively researched, prestigious and high-impact research journals worldwide, covering Science Citation Index (SCI) Social Science Citation Index (SSCI), as well as Arts and Humanities Citation Index (A&HCI) (Wouters 2006). Its vital feature is that it covers all article types, e.g., author information, institutional addresses, citations and References (Wouters 2006). Research trends and publications in specific industry areas can be effectively analyzed.

To be specific, the “Web of Science Core Collection” database is selected in Web of Science and the indexing range includes SCI, SSCI, A&HCI, CPCI-S, CPCI-SSH, BKCI-S, BKCI-SSH, ESCI databases. This step aims to expand the search scope of journals and search a maximal amount of relevant literature. A “subject search” is adopted, covering the search title, the abstract, the author and the keywords. There have been other areas of research on clothing or textiles (e.g., textile engineering and other scientific research areas). However, in this study, to ensure that the topic of analysis is relevant, the subject search is conducted by entering “fashion design” or “Costume design” clothing design”, or “Apparel design”, and only academic research regarding fashion design is analyzed. To ensure the academic nature of the collected data, the search scope here is the “article” type. The time frame was chosen from 2000 to 2021 to analyze the publications on “fashion design” for past 21 years. After this operation, the results of the search were filtered two times. The search was conducted until September 23, 2021, and 1388 articles were retained on the whole.

All bibliographic information on the pages was exported into text format and subsequently analyzed with CiteSpace software. Retrieved publications were filtered and copies were removed in CiteSpace to ensure that the respective article is unique and unduplicated in the database. 1388 articles filtered down from 2000 to 2021 were analyzed in all time slices of 1 year, and most of the cited or TOP 50 of the respective item were selected from each slice.

## Results and Discussion

### Publications in the last 21 years

**Fig. 1:**
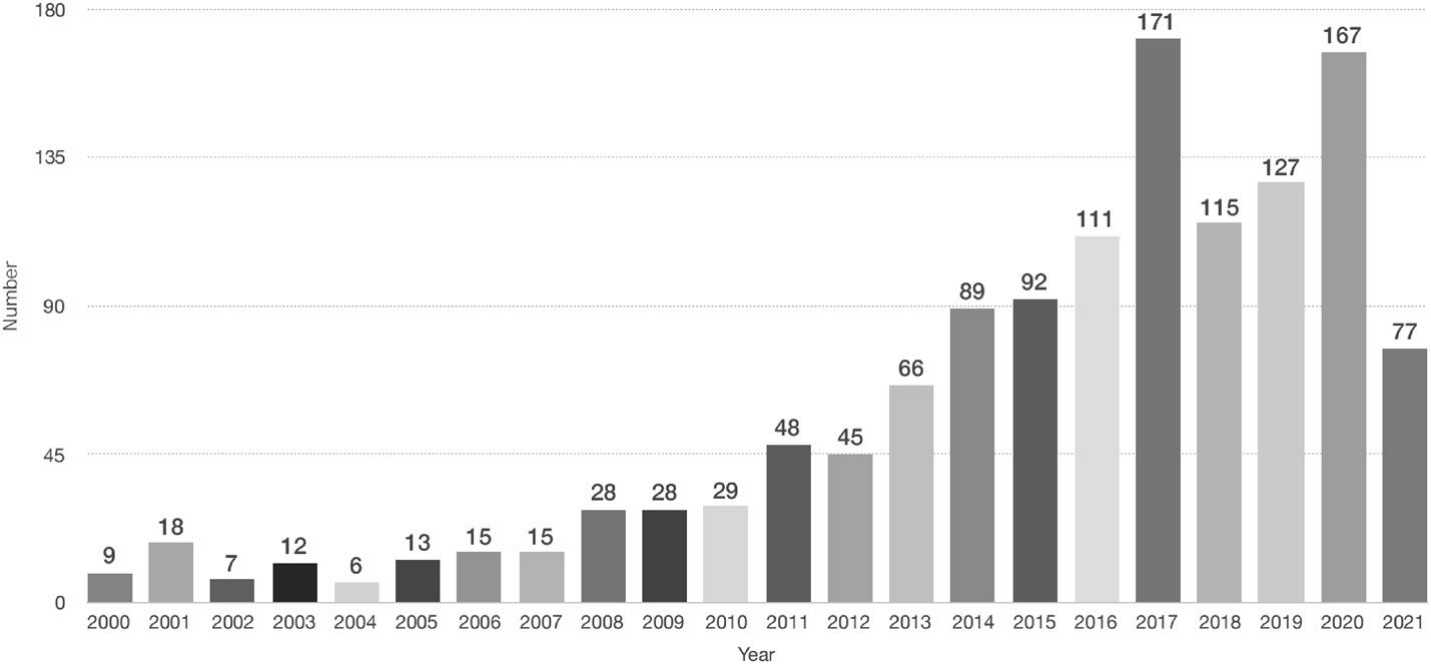
Total publications and sum of times cited from 2000 to 2021 according to the web of science. Data updated to September 2021

The publication situation of WOS database with “fashion design” as the theme from 2000 to 2021 shown in Fig. 1. On the whole, the number of articles published on the theme of “fashion design” is rising from 2000 to 2007, the number of articles published each year is almost identical, and the number of articles published in 2008–2009 is slightly increasing. The second wave of growth is in 2011, with an increase about 60% compared with the number of publications in 2010, and it has been rising year by year. 2017 is the peak year with a high volume of 171 publications. 2018 shows another decline, whereas over 100 publications remain. 2018, 2019 and 2020 show continuous growths again. As of September 2021, the number of publications in 2021 is 77. Although the number of articles declines in 2018, the overall number of articles over the past 2 decades is still rising. The reason for the low number of publications around year of 2000 is that fashion as a category of discourse has aroused the attention of scholars from the late nineteenth century (Kim, 1998). The year-on-year increase is explained as research on fashion is arousing rising attention from scholars. The significant increase in research papers regarding “fashion design” in 2016 and 2017 is that around 2016, and the fashion industry has been impacted by technological developments. Moreover, the way in which design and clothing made has incorporated considerable technological tools (e.g., 3D printing and wearable technology).

### Author co-authorship analysis

The knowledge map of cited authors based on publication references can present information regarding influential research groups and potential collaborators (Liang, Li, Zhao, et al., 2017). The function of co-authorship analysis is employed in CiteSpace to detect influential research groups and potential collaborators. Citespace can calculate the most productive authors in related fields. Table 1 shows that the most productive authors, which related with fashion design theme.

**Table 1:** Top productive authors of “fashion design”-related articles

Ranking	Count	Centrality	Year	Author	Institution and nations of Author
1	9	0.00	2015	Olga Gurova	Laurea University of Applied Sciences in Helsinki, Finland
2	7	0.00	2014	Marilyn Delong	University of Minnesota, USA
3	7	0.00	2011	Kirisi Ninimaki	Aalto University, Finland
4	6	0.00	2009	Veronica Manlow	Brooklyn College in the Koppelman School of Business, USA
5	5	0.00	2021	Caroline Kipp	The George Washington University, USA
	5	0.00	2013	Nick Reesroberts	Uk
	5	0.00	2014	Mary Alice Casto	University of Minnesota, USA
	5	0.00	2014	Kevin Almond	University of Leeds, UK
	5	0.00	2008	Hazel Clark	Parsons School of Design, USA

The author with the maximal number of publications is Olga Gurova from Laurea University of Applied Sciences in Helsinki (Finland). Her research area has focused on consumer nationalism and patriotism, identity politics and fashion, critical approach to sustainability and wearable technology and the future. Sustainable design has been a hotspot over the past decade and continues to be discussed today, and wearable technology has been a research hotpot in recent years. Olga Gurova is in first place, thereby suggesting the attention given to the mentioned topics and studies in society and fashion area. The second ranked author is Marilyn Delong from University of Minnesota (The USA). The research area consists of Aesthetics, Sustainable apparel design, History and Material Culture, Fashion Trends, Cross-cultural Influence on Design, as well as Socio-psychological aspects of Clothing. The author with the identical number of 7 publications is Kirsi Niinimäki from Aalto University (Finland). Her research directions consist of sustainable fashion and textiles, so her focus has been on the connection between design, manufacturing systems, business models and consumption habits.

For Caroline Kipp, her research area includes modern and contemporary textile arts, decorative arts and craft, craftivism, jacquard weaving, French kashmere shawls, as well as color field painting. For Nick Rees-Roberts, his research area includes fashion film, culture and digital media. Veronica Manlow from Brooklyn College in the Koppelman School of Business (USA.) The research field consists of creative process of fashion design, organizational culture and leadership in corporate fashion brands. Kevin Almond has made a contribution to creative Pattern Cutting, Clothing/Fashion Dichotomies, Sculptural Thinking in Fashion, Fashion as Masquerade. Hazel Clark, and his research field covers fashion theory and history, fashion in China, fashion and everyday life, fashion politics and sustainment. As revealed from the organization of the authors' work institutions and nations in the table, most of the nations with the maximal frequency of publications originate from the US, thereby revealing that the US significantly supports fashion design research. In general, the research scope covers fashion design, culture, mass media, craft, marketing, humanities, technology and etc. Based on the statistics of authorship collaboration, this study indicates that scholars from the US and Finland take up the top positions in the authorship publication ranking.

**Fig. 2:**
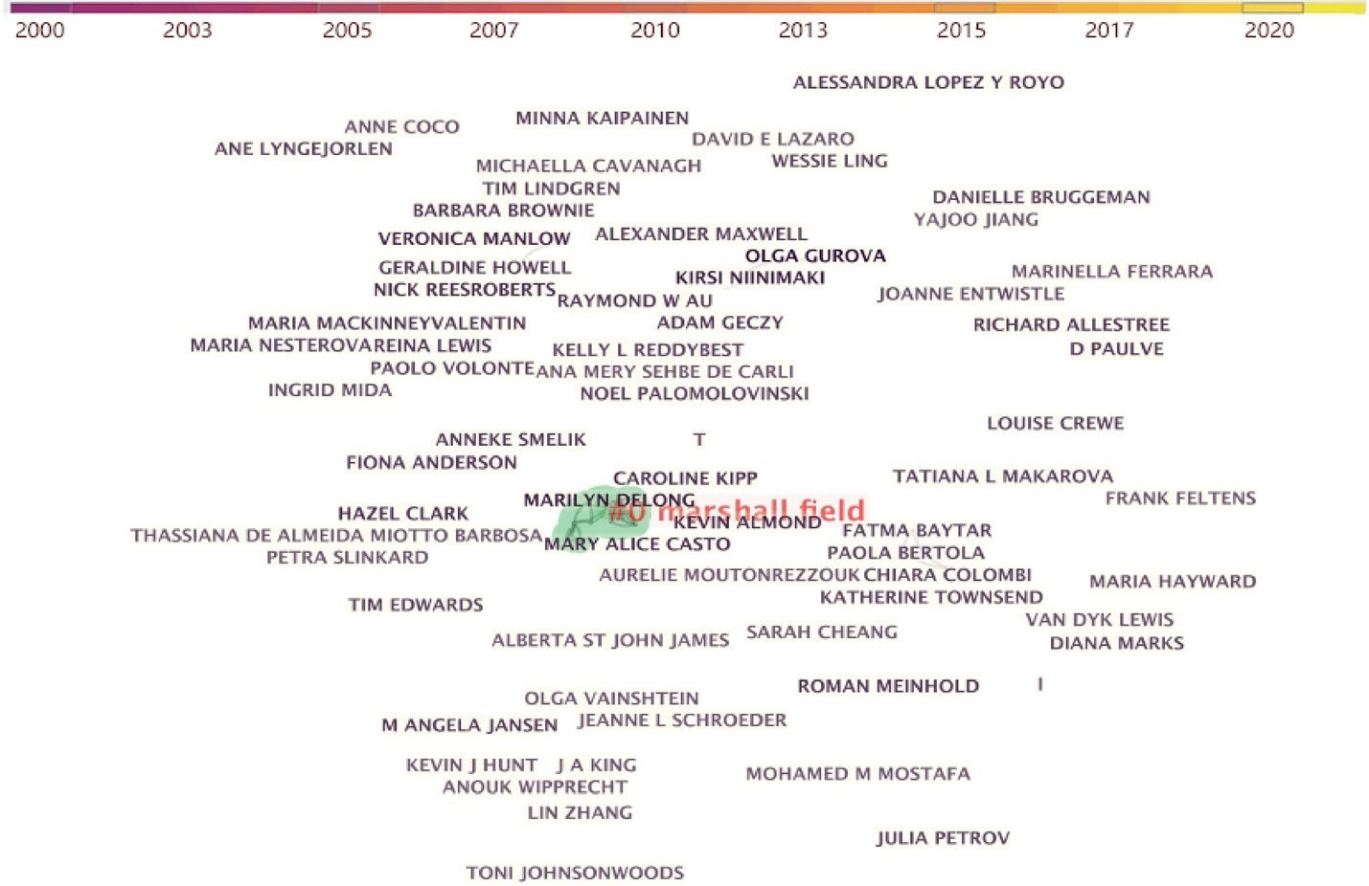
Author collaboration network

Moreover, Fig. 2 shows the academic collaborations among authors, which are generated by selecting the unit of analysis, setting the appropriate thresholds. The distance between the nodes and the thickness of the links denote the level of cooperation among authors (Chen & Liu, 2020). The influential scholars and the most active authors have not yet developed a linear relationship with each other, and collaborative networks have been lacked. It is therefore revealed that the respective researcher forms his or her own establishment in his or her own field, whereas seldom forms collaborative relationships. Thus, this study argues that to improve the breadth and depth of the field of fashion design research, the cooperation and connections between authors should be strengthened (e.g., organizing international collaborative workshops, joint publications and academic conferences) to up-regulate the amount of knowledge output and create more possibilities for fashion research.

### Institution co-authorship analysis

The number of articles issued by the respective institution and the partnership network are listed in Table 2.

**Table 2:** Top 10 productive institutions of “fashion design” related articles

Ranking	Counts	Centrality	Year	Institutions	Nations
1	22	0.00	2007	London College of Fashion	UK
2	21	0.00	2011	University Arts London	UK
3	14	0.00	2011	University of Minnesota	USA
4	13	0.00	2011	Aalto University	Finland
5	12	0.00	2013	Lowa State University	USA
	12	0.00	2008	Nottingham Trent University	UK
	12	0.00	2012	University of Leeds	UK
6	10	0.00	2016	Parsons School of Design	USA
	10	0.00	2003	University Huddersfield	UK
	10	0.00	2013	Cornell University	USA
7	9	0.00	2012	Queensland University of Technology	Australia
	9	0.00	2014	University of Brighton	UK
8	8	0.00	2010	Hong Kong Polytechnic University	China
	8	0.00	2014	Polytechinic University of Milan	Italy
	8	0.00	2017	AN Kosygin Russian State University	Russia
	8	0.00	2003	Victoria and Albert Museum	UK
	8	0.00	2013	Stockholm University	UK
	8	0.00	2010	Ryerson University	Canada
9	7	0.00	2009	Russian Acad Sci	Russia

**Fig. 3:**
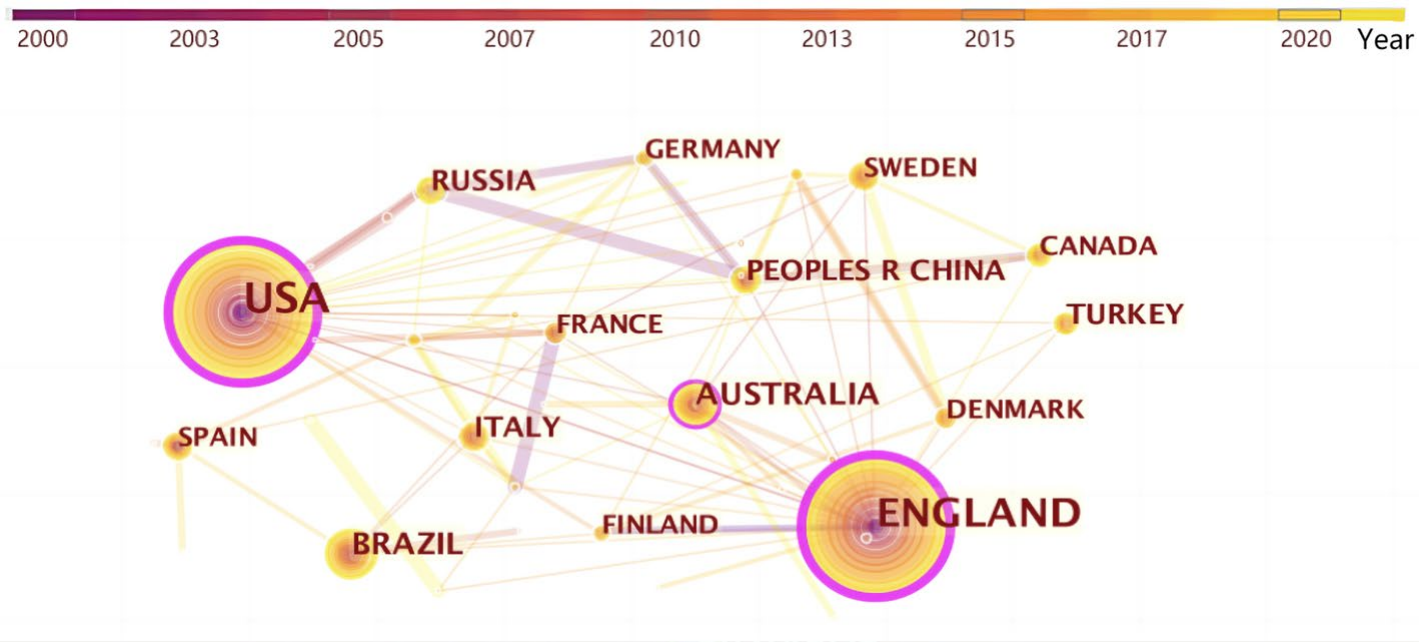
Co-relationships between nations in “fashion design” research

Figure 3 shows the collaborative relationships among research institutions, while the distance between nodes and the thickness of links represents the level of collaborative institutions. The size of the nodes represents the number of papers published by the institutions, while the distance between the nodes and the thickness of the links indicates the level of cooperation between the institutions.

As indicated from the Table 2, most of the top ten publishing institutions are from the UK and the US, and two universities in the UK rank first and second with more than 20 publications, and most of the institutions are from the UK, thereby demonstrating that the UK's achievements and effect in the field of fashion are far more than other nations. According to the cooperation network between institutions, there are 5 main cooperation networks. First, London College of Fashion, achieving the most number of articles, and there are 8 institutions cooperating with London College of Fashion, among which the closest cooperation is with Parsons Paris Sch Art & Design in the US, whose more influential areas of articles are consumers behavior, unisex clothing, third gender, communicating sustainability, real installation, Italian fashion system, global market, local culture, knitwear and textile design, international scenario, conventional craft methodologies, innovative potential, as well as 3D software application.

The second network of partnerships concentrates on University Arts London as the central node, with frequent collaborators (e.g., Center St Martins Coll Art & Design, Loughborough University Technology, Hut Grp, Sothebys Inst Art, Project Mobile Ising Sexual Hlth, De Montfort University, University of Southampton, Royal Soc Arts, Royal Coll Art). The more influential areas of publication are: electric corset, future histories, clothing sustainability, south Asian youth culture, textile patterning technique, hybrid functional clothing, UK fashion upcycling businesses, rematerializing crafting understanding, fashion designers apprentice, design ethnography approach, developing apparel design guideline and so on.

The third collaborative network is formed by Aalto University, DongHua University, University Southern Denmark, and other institutions, with more influential publications below: haring clothe; fashion designer; Chinese ethnic minority; design recipe; clothing carbohydrate binge; training design researcher; fashion design; traditional handicraft, etc.

The fourth cooperation network consists of Hong Kong Polytechnic University, Ryerson University, Queensland University of Technology, Tsinghua University, York University, Art Comm China Fashion Associate, and other institutions. The influential publications areas are: Zhongshan suit; creative application; clothing design; Chinese male; medical moment; menswear design preference; cross-national study; aesthetic aspect; evaluative criteria; disease prevention.

The fifth network organized by University Minnesota Sch Design, Seoul Natl University, Cornell University, University Calif Davis, University North Carolina Greensboro, Colorado State University and others, with the influential research areas if sustainable apparel design practice, sustainable clothing, female users’ perspective, up-cycling design process; apparel design education; strategic ambiguity effective instructional tool, as well as apparel design.

It is noteworthy that: (1) although the University of Leeds has the maximal number of papers, it has not formed a collaborative network with the University of Leeds in the analysis of collaborative relationships; UK institutions have achieved prominent research results, but in the analysis of the number of author papers, and most of the authors with more papers originate from the US. In brief, British institutions, especially university institutions, generally achieve a high level of research, whereas there are fewer authors with a particularly high number of publications. (2) Asian culture covering South Asian youth culture and Chinese fashion culture appear 3 times in the research network as one of the important research areas that combine fashion and culture. (3) Moreover, the respective sub-network has exchanges and cooperation with universities or institutions from other nations, whereas the distance between the sub-networks is long. It indicates that the self-networks have not yet formed a unified network structure among each other, and are only active within their own groups. The issuing institutions that enter the top ten are nearly universities, which acts as the main power of academic articles punishment, and few other institutions (e.g., companies or social organizations). Accordingly, the cooperation between institutions should be boosted. It is necessary to exploit their strengths and advantages, expand the research field and research scope, and make more contributions to the research on “fashion design” topic.

### Country co-authorship analysis

Table 3 lists the studies status on “fashion design” in different nations. The US has the maximal number of publications with 257 articles, followed by the UK with 226 articles.

**Table 3:** Top 10 productive countries of “fashion design” articles

Ranking	Counts	Centrality	Year	Nations
1	257	0.22	2000	USA
2	226	0.28	2000	England
3	56	0.12	2008	Australia
4	52	0.05	2007	Brazil
5	37	0.01	2009	Russia
6	36	0.02	2010	China
7	35	0.00	2001	Turkey
8	34	0.00	2010	Italy
9	31	0.03	2006	Canada
10	28	0.06	2006	Spain

**Fig. 4:**
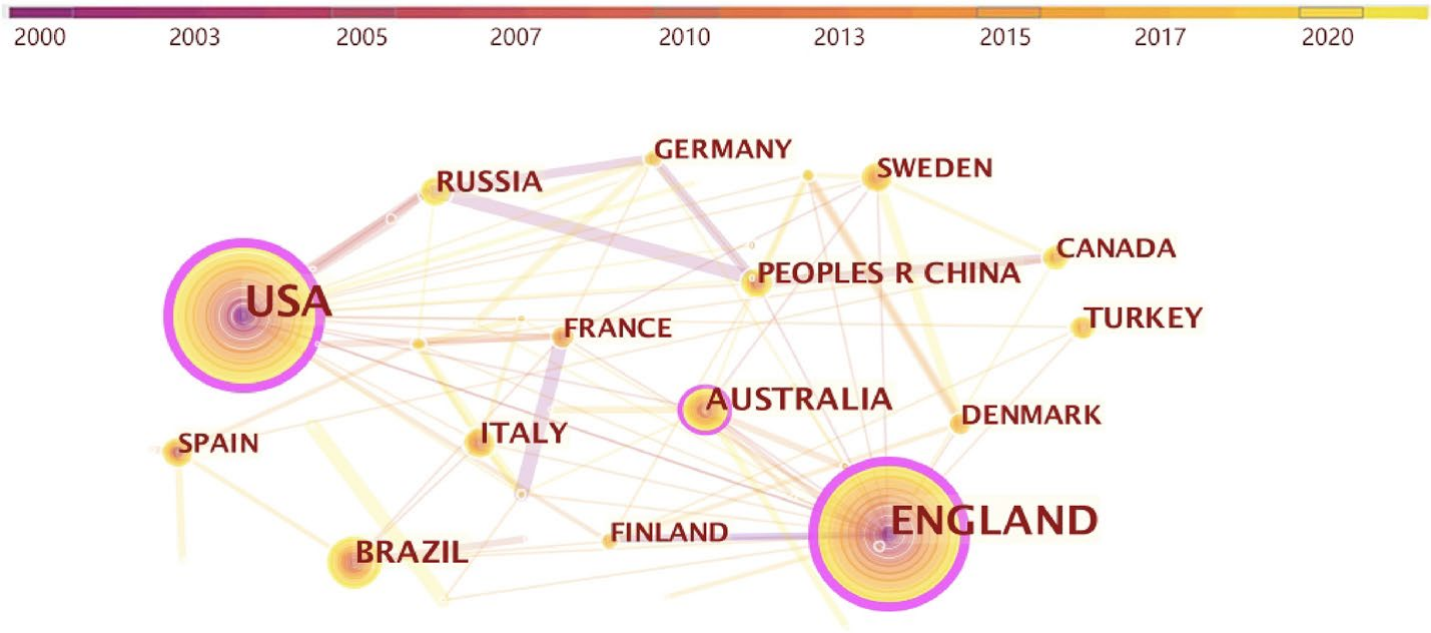
Linking relationships between nations

**Fig. 5:**
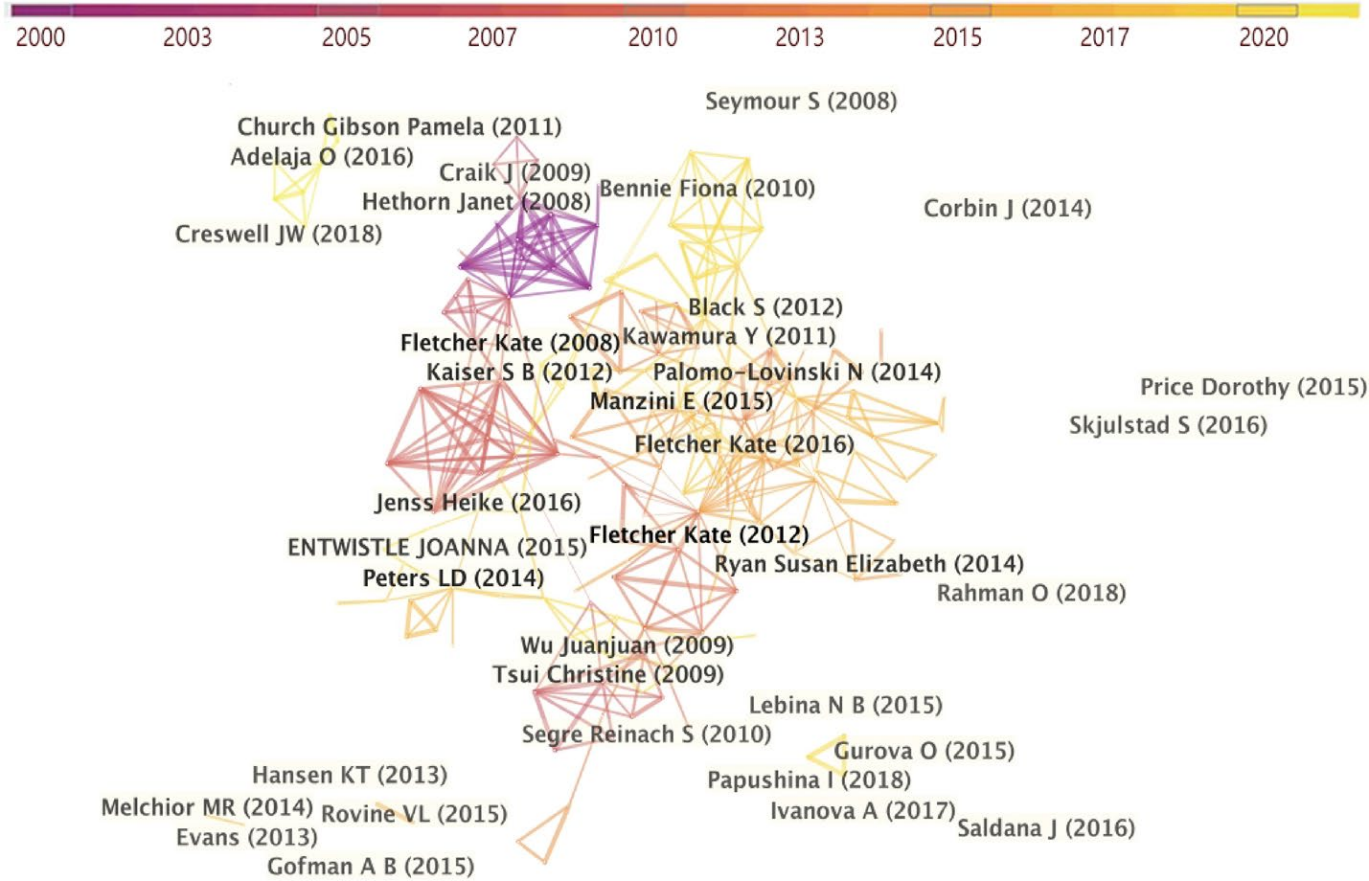
Cited references network among the literature

Figure 4 shows the co-relationships between differently nations. The nodes in the Fig. 4 represent nations, and their sizes indicate the number of articles from different nations. The distance between the nodes and the thickness of the links represents the level of cooperation between nations. The purple rings of the purple nodes indicate high centrality, which means that the mentioned nodes are key points connecting different parts of the network. The thicker the purple ring, the higher the centrality of that node. With the U.S. as the centrality degree, it links France, Lebanon, Scotland, Nigeria, Italy, Ireland, South Korea, Thailand, England, the People’s Republic of China, Sweden, Turkey, Canada, Netherlands, Finland, Switzerland, Australia, 17 nations in total. With the U.S. as the centrality degree, it links France, Italy, Brazil, Scotland, Denmark, Thailand, South Africa, Turkey, Australia, Sweden, Wales, the People’s Republic of China, North Ireland, Sweden, Canada, Netherlands, Finland, Switzerland, Germany, Egypt, America, a total of 22 nations are linked. Although the previous information on the volume of publications by institutions shows that institutions in the UK nations are dominant. However, the total volume of publications compared, the US is higher than the UK, thereby demonstrating that some other institutions or organizations besides universities also contribute to the volume of publications. Furthermore, as revealed from the degree of crossover of the cooperation network in Fig. 5, except for the UK and the US, there is but not close cooperation and connection between other nations, the nodes are far away, and the more prominent node centers are the US and the UK. This leads to the conclusion that nations should strengthen the intensity and density of cooperation and enhance their influence in fashion design research.

As indicated from the analysis of the previous network of authors, collaborating institutions and collaborating nations, the research results are more superior in the US and the UK. However, given the research statistics issued by WOW Travel in 2019, the top 10 influential nations in the field of fashion consist of the USA, the UK, Italy, France, Japan, Netherlands, Germany, Spain, the People' Republic of China, including Korea. This phenomenon is likely to be attributed to the different language systems of nations other than the UK and the US, and that some of the mentioned nations have their own search databases for articles. For this reason, the publication data are not retrieved. Other nations should actively publish in English or international academic journals to expand their effect on the international research field, not only in fashion trends or arts work creation.

### Co-scholar study based on cited references

The literature can be termed a knowledge base, as well as a source of knowledge and ideas. A novel research cannot be outputted without the contribution of knowledge from previous authors, as well as the insights into and mastery of the literature. Co-scholar analysis builds a literature co-citation network by selecting several representative studies as the object of analysis. Vital references in a specific research area can be detected, and a knowledge graph of cited authors by complying with published references can present information regarding influential research field and knowledge. Table 4 shows the most distribution of the references in fashion design theme. Figure 5 is an analysis of the highly cited literature network. Describe and summarize the high-cited literature based on the information in the two charts.

**Table 4:** Highly cited references ranking

Ranking	Counts	Centrality	Year	Cited references
1	12	0.00	2012	Fletcher Kate, 2012, Fashion sustainability
2	9	0.00	2008	Fletcher Kate, 2008, Sustainable fashion and clothing
3	8	0.00	2015	Ezio Manzini, 2012, Design, when everybody designs
	8	0.00	2014	Peters, L. D., 2014, You are what you wear: How plus-size fashion figures in fat identity formation. Fashion Theory, 18(1), 45–71
4	7	0.00	2012	SB Kaiser, 2012, Fashion and culture: cultural studies, fashion studies
5	6	0.00	2016	Heike Jenss, 2016, Fashion studies: research methods, sites, and practices
	6	0.00	2014	Susan Elizabeth Ryan, 2014, Garments of paradise: wearable discourse in the digital age
	6	0.00	2016	Fletcher, 2016, Craft of use: post-growth fashion
	6	0.00	2014	Noël Palomo-Lovinski, 2014, Fashion design industry impressions of current sustainable practices

The node density is 0.00313 for cited reference network, thereby illustrating that fewer links and co-citations among the literature. For the citation status of the respective literature, the analysis begins with the work of Fletcher Kate, appearing more frequently in the table, Fletcher Kate’s 2016 book “*Craft of use: post-growth fashion*” pertains to label 0 “fashion system”. The book explores “craft of use”, using ingenious ideas and practices to make garments/fabrics present as an alternative, dynamic, experiential framework for articulating and promoting sustainability in the fashion world (Fletcher, 2016). Fletcher, 2012 and Fletcher and Tham, 2014 pertain to the identical cluster 6. Fletcher Kate’s 2012 “*Fashion Sustainability*” counting 12 times, with ranking No.1 in Table 4. The book’s contents about fashion sustainability in three main parts, i.e., fashion products, fashion system, as well as fashion design practice (Fletcher, 2012). According to the graph, Fletcher Kate's book exhibits a high frequency in the citation network and overall citation. The book from Fletcher (2008) talking about sustainable fashion and clothing, which has the second maximal citation, frequency of 9. The book is primarily concerned with sustainable fashion and sustainable design. *Routledge handbook of sustainability and fashion,* published in 2014. The major contents focus on sustainability, and fashion recognizes the complexity of aligning fashion with sustainability. It explores fashion and sustainability at the levels of products, processes and paradigms, while employing a truly multi-disciplinary approach to critically question and suggest creative responses to issues, i.e., Fashion in a post-growth society, Fashion, diversity and equity, Fashion, fluidity and balance across natural, social and economic systems, social sciences, arts and humanities interested in sustainability and fashion (Fletcher and Tham, 2014). Fletcher Kate made prominent contributions to fashion sustainable design and sustainable development.

The third most frequently cited book is Manzini’s (2015) book "*Design, When Everybody Designs*", with eight citations. It presents Design and social Innovation, Collaborative organizations and encounters, Design ways and Design for novel cultures (Manzini, 2015). The ideas of social innovation design and sustainable design are presented.

The journal of “You are what you wear: How plus-size fashion figures in fat identity formation” from Lauren Downing Peters, takes up the third place in terms of frequency of citations. The research regarding fat identities are formed through the intimate practices of self-fashioning and via social channels (e.g., shopping and fashion blogging), thereby bridging the fields of fat studies and fashion studies. It also considers issues of performativity and is reflected as a situated bodily practice. Fashion design is combined with humanistic care (Peters, 2014).

The book Fashion and Culture: Cultural Studies, Fashion Studies, from SB Kaiser, 2012 be cited 7 times. The main topic is the integration of fashion, design and culture (Kaiser, 2012). Jenss (2016), *Fashion Studies: Research Methods, Sites, and Practices*, is cited 6 times. The book explores fashion in wide-ranging contexts by stressing material culture and ethnographic approaches in fashion studies. Ryan (2014), *Garments of Paradise: Wearable Discourse in the Digital Age,* research about the wearable fashion based on the new era (Ryan, 2014). *Fashion design industry impressions of current sustainable practices*, 2014, Noël Palomo-Lovinski, the article explores professional fashion designers' understanding and awareness of current sustainable design (Palomo-Lovinski & Hahn, 2014).

As revealed from the analysis of the co-cited literature, the literature and research areas arousing more attention in the fashion design area from 2009 to 2016 consist of fashion sustainable design and sustainable development, fashion humanities, fashion design strategies, wearable technology, fashion and culture, and Chinese fashion.

## Co-occurrence analysis for the research frontier and trends

### Hot research topics

**Fig. 6:**
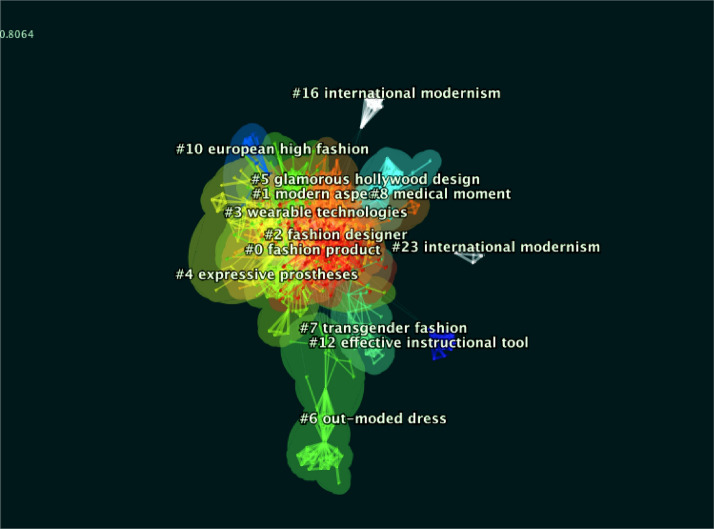
Co-citation clusters about “fashion design” theme

A research hotspot refers to a research issue or topic explored by a relatively large number of articles that are intrinsically linked within a certain period. The keywords are the authors' high distillation and summary of the core content of the article, reflecting the research value and direction of the article. Keywords achieving high frequency are generally exploited to identify the hot issues in a research field. The noun phrases extracted from the article can also represent the hotspot of research in a particular field to a certain extent. Clustering analysis of keywords is performed by CiteSpace software to generate keyword clustering knowledge graphs (Hu et al., 2019). The mentioned clusters reflect the last 21 years of topics in fashion design research (shown in Fig. 6).

The silhouette scores of the major cluster that were focused on in the review were sufficiently high. Analyzing the size of clusters by Silhouette and size, and the cluster labels could be defined by log-likelihood ratio (LLR) to explain the term contained in. The top 10 keywords in the respective cluster are summarized in the Table 5.

**Table 5:** The top 10 keywords in the respective cluster

ClusterID	Size	Silhouette	Year	Label (LLR) (Top 10)
#0 Fashion product	107	0.718	2015	Fashion product; sustainable fashion; responsible fashion business education; prolonged use; fashion entrepreneurship education; Chinese young generations perception; fashion design education; craft practice; developing nations; integrating circular economy; real installation;
#1 Modern aspect	75	0.754	2013	Modern aspect; transforming women’s fashion; early twentieth-century Russia; Chinese fashion design; Africanizing hybridity; contemporary south African fashion design; reconsidering emigre; dior closes; historic fashion houses; designer changeover
#2 Fashion designer	69	0.765	2014	Fashion designer; sustainable fashion; Chinese fashion designer; accessorizing bodyscape; different clothing; intellectual property right; milton case; haute couture; contemporary Iranian woman; aesthetics element; baluchs costume
#3 Wearable technologies	63	0.833	2014	Wearable technologies; new man; wearable art; combining ottoman miniature art; communicating identity; action film; wearing data; extended skin; new algorithmic sensibility; popular art; applied art
#4 Expressive prostheses	58	0.78	2013	Expressive prostheses; wearing class; feminine identity; postwar Australian society; month party; fashioning tradition; contemporary Korean fashion; individual experience; cultural knowledge; classic design
#5 Glamorous hollywood design	51	0.777	2014	Glamorous Hollywood design; fashion film; film costume; curatorial legacy; diana vreelands exhibition; costume drama; diana vreeland; noir side; social video; brand content;
#6 Out-moded dress	38	0.903	2007	Outmoded dress; nineteenth century; tourist attraction; hindelooper costume; historical shoe collection; shoe technology; rococo period; reliable dress source; 18th-century Neapolitan nativity scene; und schwartze kleider tragen; nuns habit dress;
#7 Transgender fashion	25	0.937	2015	Transgender fashion; dressing strategies; fit challenge; copper inuit; ceremonial clothing; empowering women; wearing plus-size clothing; using autopoiesis; ageing population; dark circus
#8 Medical moment	23	0.984	2018	Medical moment; fashioning mask; mask making; disease prevention; kuyken firm; artistic craftwork; wearables development; pattern cutting; wearable artefact; protection
#10 Glamorous hollywood design	12	0.973	2012	European high fashion; global dialogue; decorative art; enacting fashion; cultural heritage; utopian clothing; early 1920; constructivist proposal; sons experience; among father
#12 Effective instructional tool	11	0.999	2010	Effective instructional tool; enhancing spatial visualization skill; apparel design; fashion design; fashion designer; sustainable fashion; fashion industry; Chinese fashion designer; circular economy; cultural identity
#16 International modernism	9	0.999	2009	International modernism; op art; Chinese traditional Tibetan clothing; accessorizing bodyscape; costume exhibition; family album; authentic identities; fashion collection; manual operation; encountering object; fashion designer; questioning fashion
#23 International modernism	4	0.998	2007	International modernism; op art; Chinese traditional Tibetan clothing; accessorizing bodyscape; costume exhibition; family album; authentic identities; fashion collection; manual operation; encountering object

As indicated from the analysis of the keywords in the respective cluster, the research content of the respective cluster overlaps with each other. However, international research in the “fashion design” field can be summarized as eight major research fields: “Skill/Tools/Technologies/Material with fashion innovation”, “Wearing class and Art”, “Sustainable fashion”, “fashion design/ fashion designer and arts work”,” Education”, “fashion industry and business”, “fashion with culture”, “Medical fashion”.

(1) Skill/ Technologies/Materials with fashion design innovation. The common label that appears are: Wearable new materialism; technological innovation, wearable technologies, future mode, digital exploration, digital design, technological innovation, smart material systems. Promoted by the rapid development of society and science and technology, interdisciplinary learning and research has also emerged in the fashion industry. Innovative design, fabric innovation or innovative display combined with high-tech, novel materials and virtual or digital industries turns out to be a novel topic of great interest in the fashion industry (Barati, Karana, & Hekkert, 2019; Burns, 2022; Bower & Sturman, 2015; Chuah, Rauschnabel, Krey, et al., 2016; Feng, 2020; Ferrara, 2019; Huang, Tang, Liu, et al., 2018; Juhlin, 2015; Rocamora, 2017; Smelik, 2018; Smelik et al., 2016; Ünay & Zehir, 2012).

(2) Wearing culture and Arts. The common labels consist of dressing strategies, transgender fashion, men fashion, human right, accessorizing bodyscape, popular art, applied art. It focuses on different types of people, human rights and humanistic concerns, including unisex fashion. The collection is designed and worn with a mix of different arts, cultures and trends, as well as regional dress cultures, such as Chinese. The collection is inclusive of fashion and highly integrated with art (Chance, Camilleri, Winstone, et al., 2016; Geczy & Karaminas, 2011; Hancock, Johnson-Woods, & Karaminas, 2013; Martin, 1999; Mocenco, Olaru, Popescu, et al., 2016; Nelson & Hwang, 2019; Sabine Linke, 2013; Tullio-Pow, Yaworski, & Kincaid, 2021; Vainshtein, 2012).

(3) Sustainable fashion. Including the labels of sustainability knowledge, sustainable fashion, sustainable practice, communicating sustainability, sustainability knowledge, sustainable consumption, etc. Sustainable development and sustainability are a hotspot of discussion in academia. Sustainable fashion, i.e., Eco-fashion, refers to part of a growing design philosophy and sustainable design trend aiming to create a sustainable system capable of supporting environmental, socially responsible and sociocultural aspects (Aakko & Koskennurmi-Sivonen, 2013; De Brito, Carbone, & Blanquart, 2008; Fletcher, 2013; Gordon & Hill, 2015; Gwilt, 2020; Henninger, Alevizou, & Oates, 2016; Lundblad & Davies, 2016; Mukendi, Davies, Glozer, & McDonagh, 2020; Niinimäki, 2013; Shen, 2014; Wang & Lu, 2020).

(4) Fashion design, fashion designer and arts work. As the fundamental topic in fashion design field, the labels consist of design strategies, young fashion designer, costume design, South Korean contemporary fashion design, China fashion design, etc. Is the research about the characteristics of fashion in different historical stages, region, area, culture and style study (Bugg, 2009; Chang & Lee, 2021; Creigh-Tyte, 2005; Kawamura, 2004; Kim & Farrell-Beck, 2005; Larner & Molloy, 2009; Ling et al., 2016; Millspaugh & Kent, 2016; Park, 1993; Sterlacci, 2019).

(5) Education. Responsible fashion business education, teaching system, fashion design course, interactive teaching, fashion entrepreneurship education, etc. Educational methods have constantly been a vital topic required to be discussed, and teaching methods and concepts have been reformed and innovated to respond to social and economic development, as well as to the constant innovation of knowledge, skills and cultural heritage (Armstrong & LeHew, 2013; Faerm, 2012; Fletcher, 2013; Lee & Sohn, 2011; Stensaker, 2007).

(6) Fashion industry and business. In modern commodity society, the concept of fashion is more than a way of life and an inner state of mind. People's pursuit of fashion will change the existing mode of life and behavior, thereby constantly creating new demands. Accordingly, the emergence of new products is promoted, as well as the development of novel industries. Fashion products are not only characterized by commercial products, but also help create a fashion industry chain and huge economic benefits for its high added value, easy dissemination and wide circulation (Guercini & Runfola, 2010; Pal & Gander, 2018; Pedersen, Gwozdz, & Hvass, 2018; Şen, 2008; Shamsuzzoha, Kankaanpaa, Carneiro, et al., 2013; Todeschini, Cortimiglia, Callegaro-de-Menezes, & Ghezzi, 2017).

(7) Fashion with culture. The labels include cultural heritage, traditional craft methodologies, new vision, cultural identity, cultural knowledge, etc. Understanding the effect of culture on the fashion industry and design creation gives insight into the style of fashion people want. For the identical reason, fashion impacts the way we live. Fashion is impacted by changes in culture (e.g., modernization, art, and even innovative technology). It is noteworthy that fashion is created by people living in different cultures and places. If one wants to understand fashion, one should be aware of the cultures of different places (e.g., traditional cultures, cultural heritage, new cultural contexts, and cross-cultural exchanges) (Fillin-Yeh, 2001; Jansen, 2014; Ko & Lee, 2011; Roche, 1996; Rocamora, 2017; Woodside & Ko, 2013; Zou and Joneurairatana, 2020a, b).

(8) Medical fashion, the labels (e.g., mask making, world view, disease prevention, wearable development and fashioning masks). The medical area fashion is listed as a separate field because of the specificity and timing of this field. Since the outbreak of Covid-19 in 2020, the concern for health and disease worldwide has become an essential topic, and almost every research area has a connection with medical care, as impacted by such a general trend and environment, led to developments in the field of “medical care fashion” (e.g., the development of new materials, masks and protective fashion). In addition, due to the development of “human centred design” thinking, the current fashion industry not only pays attention to the creation of artistic works, but also pays more attention to humanistic care. The needs of special groups have also attracted the attention of the fashion industry, such as disabled people, etc. (Kim, et al., 2021; Koenig & Carnes, 1999; Li & Yim, 2021).

### Keywords with the strongest citation bursts

Keywords with the strongest citation Bursts can be exploited to reflect the main research content of a research topic over time, and also to reflect the research trends in a certain time period. The tracking and identification of research trends can offer researchers information regarding the changes in research hotspots in the field of specialization, and can provide relevant inspiration and information for researchers in the field. Research frontiers are emerging theoretical trends and new topics that can be synthesized and judged in CiteSpace based on analysis of keywords with the strongest citation bursts (Li & Wang, 2018).

**Fig. 7:**
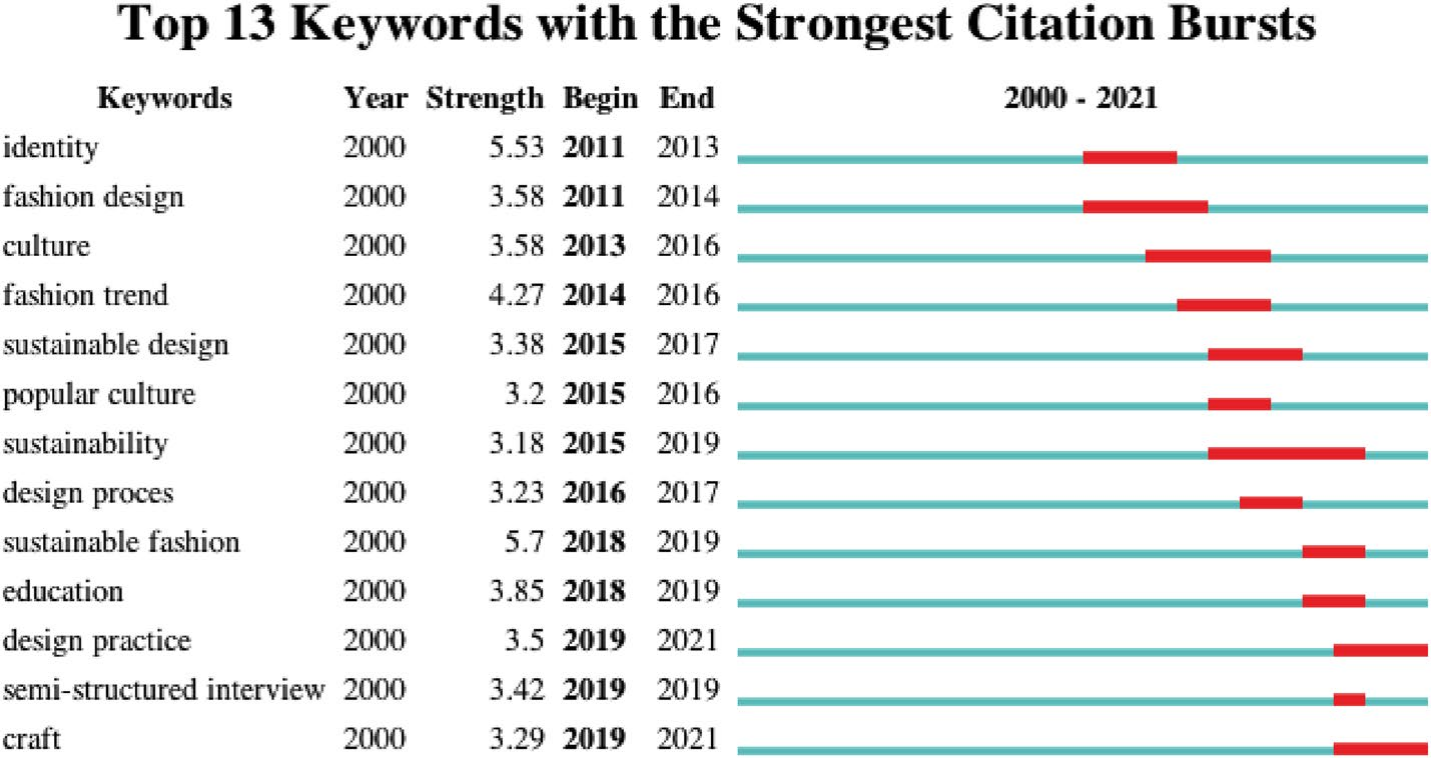
Top 13 keywords with the strongest citation bursts in “fashion design” area

After running the CiteSpace software, 13 keywords with maximal citation bursts were obtained (shown in Fig. 7).

In this study, the research scope is selected from 2000 to 2021, and the strongest citation bursts are concentrated after 2010. The mentioned consist of identity, culture, fashion trend, popular culture, design process, education, design practice, craft, etc. Moreover, the analysis of the strongest citation bursts complies with the following noteworthy points:

The topic of sustainable fashion has burst on the scene three times over the last decade, i.e., in 2015 for “sustainable design”, in 2015 for “sustainability”, as well as in 2018 for “sustainable fashion”. The evolution of sustainable fashion can be identified in the shift from “sustainable production” to “sustainable fashion” concepts. In the wake of the world's biggest ever garment industry disaster, the collapse of the Rana Plaza factory in Bangladesh, having caused death of over 1100 people (Rahman, 2014) the fashion movement by complying with the concept of “sustainability” is fading massively, which reveals an increased interest in sustainable fashion and ethical practices in the fashion industry (Westervelt, 2015). As sustainability turns out to be a “megatrend” (Mittelstaedt, Shultz, Kilbourne, et al., 2014), the fashion field has changed dramatically in accordance with the concept of “sustainable fashion” (e.g., sustainable design, fabrics, production and consumption) (Watson & Yan, 2013; Mora et al., 2014). Moreover, today sustainable fashion refers to a movement and process facilitating the transformation of fashion products and fashion systems towards greater ecological integrity and social justice. Sustainable fashion is not only concerned with fashion textiles or products, but concerned with the dependent social, cultural, ecological and financial systems correlated with people.

### Research rends and frontier on fashion design

The identification and tracking of research frontiers present researchers with the latest developments in the disciplinary research evolution, predicts the trends in the research field, and identifies issues required to be explored more specifically. Research frontier topics are novel topics of interest in the field, indicating the social environment and research context. In brief, it can be referenced for relevant researchers in this field.

**Fig. 8:**
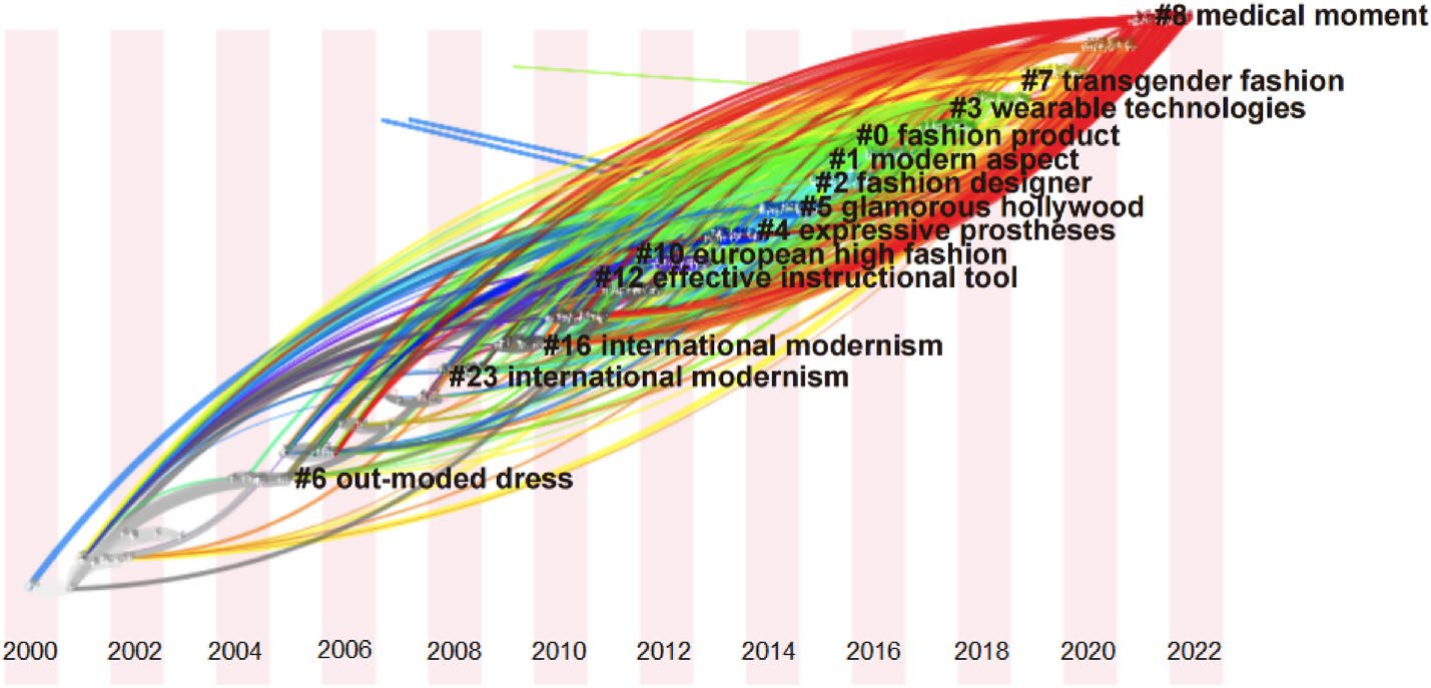
Time zone view in fashion design research

After CiteSpace is run, keyword timing profiles are generated by time segment based on Cluster co-occurrence analysis (shown in Fig. 8).

From the time zone view, the research in fashion design can fall into four phrases. The first phrase is that the research situation before 2004 did not form a cluster, thereby indicating that the research on fashion design was scattered before 2004. The second phrase is from 2004 to 2010, thereby revealing that the term of “international modernism” appeared twice. It can be explained by the frequent cross-cultural exchange activities between nations. The research emphasis shifts from fashion research in the traditional sense (e.g., apparel characteristics, designer and design styles) to cross-cultural and regionally fashion culture research (e.g., China, Europe, and the US). The third phase is from 2011 to 2017, more clusters appear in this time period, thereby demonstrating a higher volume of articles published. The research topics in fashion design show a diversity of clusters keywords and a wider range of research directions (e.g., culture, regional fashion, traditional apparel, humanities, education, design approaches and techniques). The fourth stage is from 2017 to the present, the keywords of clusters are more obvious, especially the label around 2017: “wearable technologies”. The mentioned keywords include wearable technology, wearable devices, fashion technology, smart wear, and technology socks. This novel technology is “skin electronics” or “fashion electronics”, which are intelligent electronic devices worn near or on the skin surface to detect, analyze and transmit information regarding the body information, body signals, vital signs or environmental data and others; in several cases, the information can be delivered to the wearer (Chuah, Rauschnabel, Krey, et al., 2016; Çiçek, 2015; Farrington, 2016). The second label is “Transgender Fashion”, unisex fashion, embodies the humanistic nature of fashion. Moreover, the label in 2021 is concerned with “Medical Moment”. With the global outbreak of Covid-19, how to against the virus is the daily topic be concerned by global. Protective clothing, mask has become a necessity in people's lives. Based on this context, the fashion industry has also been affected. The fashion industry think more about the care and needs of the human body, “Medical fashion” has become a popular topic of research. As indicated from the academic view, the research direction of fashion design is closer to the society hot trends and interdisciplinary research. Caring for people's physical, physiological and psychological aspects, fashion research tends to be more human centred design.

## Conclusions

By analyzing the frontiers and trends of fashion design research, this study reveals that at the beginning of the research period, the topics of academic research were biased towards research in the humanities (e.g., fashion design, designers, culture, humanistic care, locality, as well as arts work). The direction of research over the past few years has been impacted by the overall global dynamics as well as technological and economic development, thereby demonstrating that the trend of interdisciplinary and cross-border cooperation has entered a stage of development in recent years. The data collection and analysis time of this article is at the end of 2021, but with the development of time and science and technology, such as Digital fashion, Virtual fashion, AI design, Inclusive design, etc. have also become hot topics at the moment. The researcher believe it will produce more academic research in fashion design in the future time.

On the whole, research on the topic of fashion design still has a considerable scope for research. Scholars, designers and practitioners in the fashion field still face huge task. Accordingly, the researcher proposed several suggestions for how to strengthen the process and results of academic research. From a horizontal perspective, (1) the international academic community and researchers should enhance the interact, discuss and conduct collaborative research with each other to provide sustainable vitality and motivation for the research; (2) transnational, cross-unit and cross-border academic exchange and cooperation should be enhanced to create more possibilities for academic research; (3) additional, multilingual journal platforms should be offered for fashion or art fields. From vertical perspective: Combining or contrasting history with modernity. For instance, using new technologies to redesign or study historical apparel, etc. By combining traditional culture with modern technology, the scope of the time-line of fashion design research can be extended.

This study uses quantitative literature analysis to convey information from the literature by creating images, diagrams and information description. The existing state of research in fashion design is reviewed, and provide the knowledge base, the existing state of research, as well as research hot-spots and publication trends in fashion design research. This study can provide existing literature, knowledge map, new inspirations, and research directions to fashion practitioners, researchers, and research institutions. Based on this paper, scholars can efficiently familiarize the field knowledge and facilitate strategic adjustments by relevant institutions.

## Data Availability

The datasets supporting the research process and conclusions of this article are included within the additional files. For databases and research results, which is available and has no restrictions to its use by academics or non-academics.
